# C-reactive protein enhances activation of coagulation system and inflammatory response through dissociating into monomeric form in antineutrophil cytoplasmic antibody-associated vasculitis

**DOI:** 10.1186/s12865-015-0077-0

**Published:** 2015-03-03

**Authors:** Peng-cheng Xu, Shan Lin, Xiao-wei Yang, Dong-mei Gu, Tie-kun Yan, Li Wei, Bao-li Wang

**Affiliations:** Department of Nephrology, Tianjin Medical University General Hospital, Tianjin, 300052 China; Department of Nephrology, Shandong Provincial Hospital affiliated to Shandong University, Jinan, Shandong 250021 China; Department of Clinical Laboratory, Tianjin Medical University General Hospital, Tianjin, 300052 China; Key Lab of Hormones and Development (Ministry of Health), Metabolic Diseases Hospital & Tianjin Institute of Endocrinology, Tianjin Medical University, Tianjin, 300070 China

**Keywords:** C-reactive protein, Antineutrophil cytoplasmic antibody, Vasculitis, Platelets

## Abstract

**Background:**

C-reactive protein (CRP) exerts prothrombotic effects through dissociating from pentameric CRP (pCRP) into modified or monomeric CRP (mCRP). However, although the high prevalence of venous thromboembolism (VTE) in patients with anti-neutrophil cytoplasmic antibody (ANCA)-associated vasculitis (AAV) has been identified, it remains unclear whether the high levels of circulating pCRP potentially contribute to this hypercoagulable state in AAV. ANCA can induce the generation of neutrophil extracellular traps (NETs). In this study, the NETs-dependent generation of mCRP from pCRP and the influences of mCRP on the activation of coagulation system and inflammatory response in AAV were investigated.

**Results:**

NETs were induced after TNF-α primed neutrophils were incubated with ANCA-containing IgG. After ANCA-induced netting neutrophils were incubated statically with platelet-rich plasma (PRP) containing mCRP (60 μg/mL), the proportion of platelets expressing CD62p increased significantly, while no increased CD62p expression of platelets was observed after static incubation with PRP containing pCRP (60 μg/mL). Under flow conditions, perfusing immobilized ANCA-induced netting neutrophils with pCRP-containing PRP caused platelets activation and mCRP deposition. The newly generated mCRP induced platelets activation on ANCA-induced netting neutrophils, enhanced D-dimer formation, and enhanced high mobility group box 1 secretion by platelets.

**Conclusions:**

Under flow conditions, ANCA-induced netting neutrophils can activate platelets and then prompt the formation of mCRP on activated platelets. Then the newly generated mCRP can further enhance the activation of platelets, the process of thrombogenesis, and the inflammatory response. So the high level of circulating pCRP is not only a sensitive marker for judging the disease activity, but also a participant in the pathophysiology of AAV.

## Background

Although anti-neutrophil cytoplasmic antibody (ANCA)-associated vasculitis (AAV) is a systemic autoinflammatory disorder that affects small vessels and capillaries predominantly, it had been observed in a number of studies that there is high prevalence of venous thromboembolism (VTE) in patients with AAV [1-6]. The levels of circulating D-dimer, a fibrin degradation product after a blood clot is degraded by fibrinolysis, are significantly higher in AAV patients in active stage compared with those in remission [7]. However, the underlying mechanism of this hypercoagulable state in AAV is not yet fully clarified.

The level of circulating C-reactive protein (CRP) is elevated in active phase of AAV, and falls rapidly with remission of the disease [8]. Interestingly, Ma et al. recently found that AAV patients with elevated circulating D-dimer had more severe clinical manifestations and higher levels of circulating CRP compared to patients with normal circulating D-dimer. Moreover, correlation analysis showed that the levels of circulating D-dimer positively correlated with the levels of circulating CRP [7]. Circulating CRP belongs to the pentraxin family and contains 5 identical, noncovalently linked subunits (pentameric CRP, pCRP). Many studies have demonstrated that in cardiovascular disease, the circulating pCRP can dissociate into modified or monomeric CRP (mCRP) on activated platelets and accelerate thrombogenesis [9-12]. Compared with cardiovascular disease, the circulating pCRP levels in active AAV are generally much higher, but its significance in VTEs of AAV had been less well studied.

In AAV, ANCA can induce the neutrophil extracellular traps (NETs) formation after binding neutrophils. NETs are DNA fibers comprising histones, antimicrobial proteins and tissue factor [13-17]. Since some previous studies have reported that NETs can provide a scaffold for platelets adhesion and activation, it is reasonable to hypothesize that circulating pCRP can transform to mCRP on activated platelets by the aid of ANCA-induced netting neutrophils and the newly generated mCRP can accelerate thrombogenesis in AAV. On the other hand, cumulative evidences have proved a biologic association between thrombogenesis and inflammation. Elevated inflammation is both a cause and a result of thrombogenesis [18,19]. Thus, it is also reasonable to speculate that circulating CRP not only takes part in the process of thrombogenesis in AAV, but also enhances the subsequent inflammatory response following VTEs. High mobility group box 1 (HMGB-1) is an intracellular protein that can bind DNA and regulate gene expression. Besides, it can also function as a kind of proinflammatory mediator when released from cells [20]. Recently published studies have demonstrated that the activated platelets can secrete HMGB-1 [21,22].

In this study, we investigated whether ANCA-induced netting neutrophils could activate platelets and then prompt the formation of mCRP on activated platelets. We also investigated whether mCRP could further enhance the activation of platelets, the formation of D-dimer and the secretion of HMGB-1 of platelets.

## Methods

### Patients

Patients’ plasma was obtained from 5 AAV patients with positive myeloperoxidase (MPO)-ANCA. All 5 patients, diagnosed in Tianjin Medical University General Hospital, fulfilled the 2012 revised International Chapel Hill Consensus Conference Nomenclature of Vasculitides [23]. Plasma from 2 healthy blood donors was obtained as normal control. The research was in compliance of the declaration of Helsinki and approved by the Ethics Committee of Tianjin Medical University General Hospital. All participants have provided their written informed consent.

### Preparation of materials

High purity (>99%) human pCRP obtained from plasma was purchased from Sigma-Aldrich (C-4063; St. Louis, MO USA). pCRP was saved in buffer containing 5 mM calcium chloride. The level of endotoxin was undetectable as determined by the Limulus amebocyte lysate assay (ET0200, Sigma-Aldrich). mCRP was generated by treating pCRP with 8 M urea in the presence of 10 mM EDTA for 1 hr at 37°C as described previously [24]. IgG fractions from patients with positive ANCA or healthy donors were purified by protein G affinity column (GE Healthcare Life Sciences) and combined together in the following experiments.

Neutrophils were isolated by density centrifugation using a Histopaque gradient. Briefly, a double gradient was formed by layering an equal volume of 3 mL Histopaque-1077 (10771, Sigma-Aldrich) over 3 mL Histopaque-1119 (11191, Sigma-Aldrich). Ethylene diamine tetraacetic acid (EDTA) anti-coagulated whole blood (6 mL) was carefully layered onto the upper Histopaque-1077 medium. After 30 min centrifugation at 700 g, neutrophils between two Histopaque medium were carefully isolated. Cells were washed by addition of 5 mL of isotonic phosphate buffered saline (PBS) (0.20 g/L of KCl, 0.20 g/L of KH_2_PO_4_, 8 g/L of NaCl, 1.15 g/L of Na_2_HPO_4_) to the tubes. Purity of neutrophils was >90% as assessed by flow cytometric analysis.

Platelet-rich plasma (PRP) was obtained from citrate anti-coagulated whole blood by centrifugation at 20 g for 15 min. The concentration of platelets was adjusted to 100 × 10^9^/L. To remove platelets, PRP was centrifugated at 3000 g for 15 min and then platelet-free plasma (PFP) was obtained.

### Induction of netting neutrophils by ANCA and isolation of NETs

Neutrophils (1 × 10^6^/mL) were primed with 5 ng/mL TNF-α (H8916, Sigma-Aldrich) and incubated with ANCA-containing IgG (250 μg/mL) purified from AAV patients at 37°C for 4 hr. To collect NETs, the ANCA-containing supernatant was removed after centrifugation at 3000 g for 5 min. The precipitate was resuspended with PBS and was agitated vigorously for 3 min. Then NETs were collected in the supernatant after centrifugation at 200 g for 5 min. NETs quantity were assessed by measuring concentrations of dsDNA with MaestroNano Micro-Volume Spectrophotometer (MN-913, Maestrogen Inc., Hsinchu, Taiwan).

### Flow cytometry

PRP was incubated with ANCA-induced netting neutrophils (1 × 10^6^/mL), pCRP (60 μg/mL), mCRP (60 μg/mL), 0.1 U/μL DNase I (D7076, Beyotime Institute of Biotechnology, Haimen, China), netting neutrophils plus pCRP, netting neutrophils plus mCRP, or netting neutrophils plus mCRP and DNase I at 37°C for 1 hr respectively. Adenosine diphosphate (ADP) (A2754, Sigma-Aldrich) at a concentration of 100 μmol/L was used as a positive control. A fluorescein isothiocyanate (FITC) conjugated anti-CD41a mAb (340929, BD, USA) at a dilution of 1: 10 was used as an activation-independent marker of platelets. CD62p was assessed with a phycoerytrin (PE) conjugated anti-CD62P mAb (561921, BD, USA) at a dilution of 1: 10. After PRP was incubated with antibodies at 37°C for 30 min, platelets were centrifuged, washed, resuspended and assessed by flow cytometry analysis.

### Perfusion experiment and colocalization of platelets and mCRP on ANCA-induced netting neutrophils

Neutrophils (1 × 10^6^/mL) were coated on the Poly-L-Lysine-coated slides in PBS at 37°C for 1 hr and then were treated with 5 ng/mL TNF-α (H8916, Sigma-Aldrich) and ANCA-containing IgG (250 μg/mL) at 37°C for 4 hr. Nonspecific binding sites were blocked in PBS containing 2% bovine serum albumin (BSA). The slides were placed in flow chambers (31–010, Glycotech, USA) and perfused with 10 mL citric acid-anticoagulated PRP or PFP for 10 min at constant shear rates of 1500 s^−1^. Where indicated, pCRP (60 μg/mL) or mCRP (60 μg/mL) was added to PRP or PFP before perfusions. After perfusions, slides were rinsed with PBS and fixed with 3.8% paraformaldehyde.

Netting neutrophils were stained using 4’,6-diamidino-2-phenylindole (DAPI) (C1006, Beyotime). Activated platelets were visualized using FITC conjugated anti-CD62p mAb (550866, BD, USA) at a dilution of 1: 10. mCRP was identified using specific mouse monoclonal anti-C-reactive protein (Clone CRP-8) (C1688, Sigma-Aldrich) at a dilution of 1: 2000 and tetraethyl rhodamine isothiocyanate (TRITC)-goat anti-mouse antibodies (T5393, Sigma-Aldrich) at a dilution of 1: 50. The results were visualized with fluorescence microscopy (Leica DMI4000 B) and evaluated with the Image Pro Plus analysis software 6.0.

To investigate the effect of mCRP deposited on the activated platelets during perfusion experiments on the activation of platelets, ANCA-induced netting neutrophils were perfused with PRP containing pCRP or mCRP as mentioned above. The activated platelets on slides were visualized with FITC conjugated anti-CD62p mAb (550866, BD) and calculated as activated platelets/visual field.

### Western-Blot analysis

Polyacrylamide gel electrophoresis (PAGE) with 1/20 of normal levels of sodium dodecyl sulfate (SDS) was performed as described previously with minor modifications [23]. Concentration of stacking gel and separation gel was 6% and 8% respectively. Prestained molecular mass markers (SM1811, Thermo Scientific Fermentas) were used to identify the molecular mass of proteins. pCRP and mCRP were loaded as controls (1 μg per lane). In order to investigate whether the pCRP or mCRP was deposited after perfusions, substances on the slides after perfusion experiments were washed off with 200 μL Hanks solution (containing 1.26 mmol/L Ca^2+^) and were loaded (20 μL per lane). In order to investigate whether NETs could directly dissociate pCRP into mCRP, pCRP was incubated with isolated NETs at 37°C for 12 hr in Hanks solution (containing 1.26 mmol/L Ca^2+^) and then were loaded (1 μg per lane). Electrophoresis was performed for 60 min. After electrophoresis, the proteins on gel were transferred onto nitrocellulose membrane at 0.08 mA/cm^2^ for 70 min and blocked overnight at 4°C in Tris buffered saline with Tween-20 (TBST) [0.01 mol/L Tris–HCl, pH 7.2, 0.15 mol/L NaCl, 0.1% (v/v) Tween 20] containing 1.0% BSA. The membrane was incubated with Clone CRP-8 (C1688, Sigma-Aldrich) diluted 1:20000 in TBST containing 1.0% BSA for 1 hr at 37°C. After triple wash with TBST (10 min each), the membrane was incubated with horseradish peroxidase-conjugated goat anti-mouse antibody (136815, Abcam) diluted 1:10000 in TBST containing 1.0% BSA for 1 hr at 37°C. The result was revealed on autoradiographic film using ECL Plus Western Blotting Detection System.

### Enzyme-linked immunosorbent assay (ELISA) for analyzing the binding between mCRP and isolated NETs

Isolated NETs with the adjusted dsDNA concentration of 5 μg/mL were coated on the Poly-L-Lysine-coated plates in PBS at 37°C for 1 hr. Nonspecific binding sites were blocked in PBS containing 2% BSA at 37°C for 1 hr. mCRP at 4.0 μg/mL in PBS was added to the wells in duplicate and incubated at 37°C for 1 hr. Where indicated, 0.1 U/μL DNase I (D7076, Beyotime) was added to wells after NETs were coated on plates, or 0.1 mg/mL polyclonal rabbit anti-MPO antibodies (N5787, Sigma-Aldrich) were added to wells before mCRP was added at 37°C for 1 hr. Then Clone CRP-8 (C1688, Sigma-Aldrich) diluted 1: 1000 in PBS was added at 37°C for 1 hr followed by incubation at 37°C for 1 hr with anti-mouse IgG (Fab specific)–alkaline phosphatase antibody produced in goat (A1293, Sigma-Aldrich) diluted 1:2000 in PBST. The p-nitrophenyl phosphate (N2770, Sigma-Aldrich) at a concentration of 1 mg/mL was used in substrate buffer [1 M diethanolamine and 0.5 mM MgCl_2_ (pH 9.8)]. Color development was measured spectrophotometrically at 405 nm (Bio-Rad, Tokyo, Japan). In each step, the volume was 100 μL and the plates were washed three times with PBS between steps. All samples were tested in duplicate.

### Measurement of the levels of D-dimer

D-dimer levels of the samples were analyzed on the VIDAS system (bioMérieux) in our central laboratory. The single-dose, ready-to-use reagent included a solid-phase receptacle and a strip. 200 μL of samples were added to the first well on the strip and then inserts the strip and corresponding solid-phase receptacle into the analyzer. The whole assay is carried out at 37°C for 35 min. The D-dimer concentration was calculated from a calibration curve specific to the reagent batch stored in the software.

### Measurement of the levels of high-mobility group box 1 (HMGB-1)

Plasma levels of HMGB-1 were tested using commercially available ELISA kits (Shino-TEST). The assay was conducted according to the manufacturer’s instructions. The specimen (10 μL) was added to immobilized anti-HMGB-1 antibody on the well together with 100 μL sample diluent, allowing HMGB-1 to specifically bind to the antibody. Peroxidase-conjugated secondary antibody was then added to the sample well. Chromogenic solution was added to the antigen-antibody complex for assaying absorbance at 450 nm.

### Statistical analysis

Different conditions were performed at least three times in each subject. Data were shown to be normally distributed using the Kolmogorov-Smirno test and variables were expressed as mean ± SD and were evaluated using independent t-test or one-way ANOVA analysis as appropriate. It was considered significant difference if the P-value was less than 0.05. Analysis was performed with SPSS statistical software package (version 19, Chicago, IL, USA).

## Results

### Platelets are activated after static incubation with mCRP or DNase I-pretreated netting neutrophils

**Figure 1:**
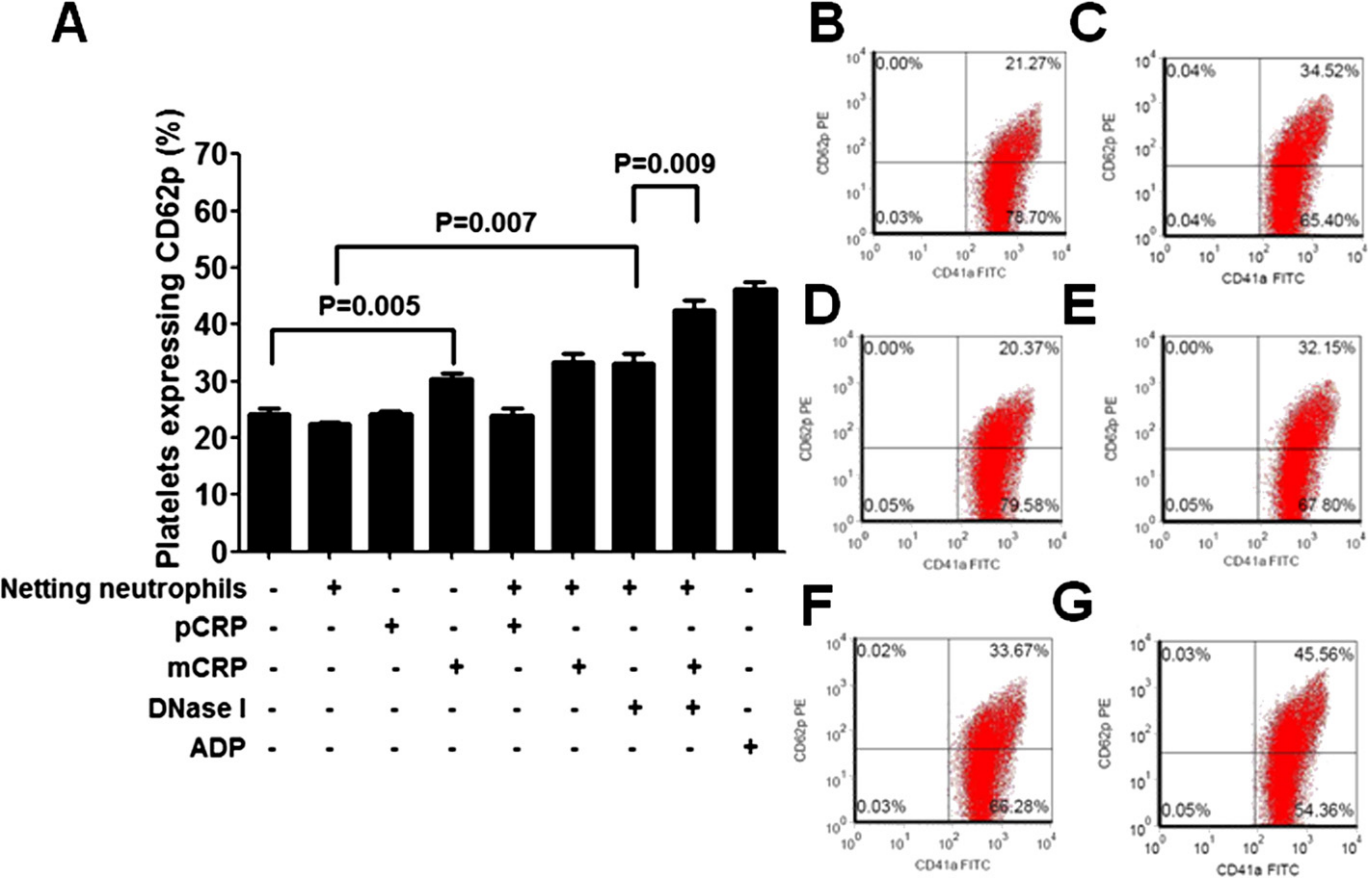
**CD62p expression of platelets in static incubation system. A**: Flow cytometry analysis of the effect of pCRP and mCRP on the CD62p expression of platelets incubated statically with ANCA-induced netting neutrophils. ADP at a concentration of 100 μmol/L was used as a positive control. Results are expressed as the percentages of CD62p positive platelets. Four tubes of each group were analyzed and averaged. **B-G**: Representative images of the flow cytometry analysis of the percentages of platelets expressing CD62p. B: PRP were incubated with ANCA-induced netting neutrophils alone. **C**: PRP were incubated with ANCA-induced netting neutrophils pretreated with DNase I (0.1 U/μL). **D**: PRP were incubated with pCRP (60 μg/mL) alone. **E**: PRP were incubated with mCRP (60 μg/mL) alone. **F**: PRP were incubated with ANCA-induced netting neutrophils and mCRP (60 μg/mL). **G**: PRP were incubated with ANCA-induced netting neutrophils, mCRP (60 μg/mL) and DNase I (0.1 U/μL).

When TNF-α primed neutrophils were incubated with ANCA-containing IgG for 4 hr, DAPI staining showed that NETs formation could be observed in about 20% of neutrophils, while normal IgG did not induce NETs formation. Static co-incubation of PRP and ANCA-induced netting neutrophils did not increase the CD62p expression of platelets (22.38 ± 0.72% *vs.* 24.23 ± 1.98% of untreated PRP, p = 0.131), while co-incubation of PRP and ANCA-induced netting neutrophils which were pretreated with DNase I increased the CD62p expression of platelets up to 33.20 ± 3.42% (p = 0.007, compared with the CD62p expression of platelets after co-incubation of PRP and ANCA-induced netting neutrophils). These results suggested that the digested NETs could activate platelets in static condition. It should be explained that the digestion of DNA exposed histones which could activate platelets directly [16]. Co-incubation of PRP, DNase I-pretreated netting neutrophils and mCRP could increase the CD62p expression of platelets up to 42.48 ± 1.71% (P = 0.009, compared with the CD62p expression of platelets after co-incubation of PRP and DNase I-pretreated netting neutrophils). Incubation of PRP with mCRP alone also increased the CD62p expression of platelets (30.45 ± 2.02% *vs.* 24.23 ± 1.98% of untreated PRP, p = 0.005), while incubation of PRP with pCRP alone did not (24.32 ± 0.62% *vs.* 24.23 ± 1.98% of untreated PRP, p = 0.930). So mCRP could activate platelets in static condition. Incubation of PRP with ADP (100 μmol/L) which was used as a positive control also increased the CD62p expression of platelets significantly (Figure 1).

### Perfusing ANCA-induced netting neutrophils with pCRP-containing PRP induces the formation of mCRP on activated platelets

**Figure 2:**
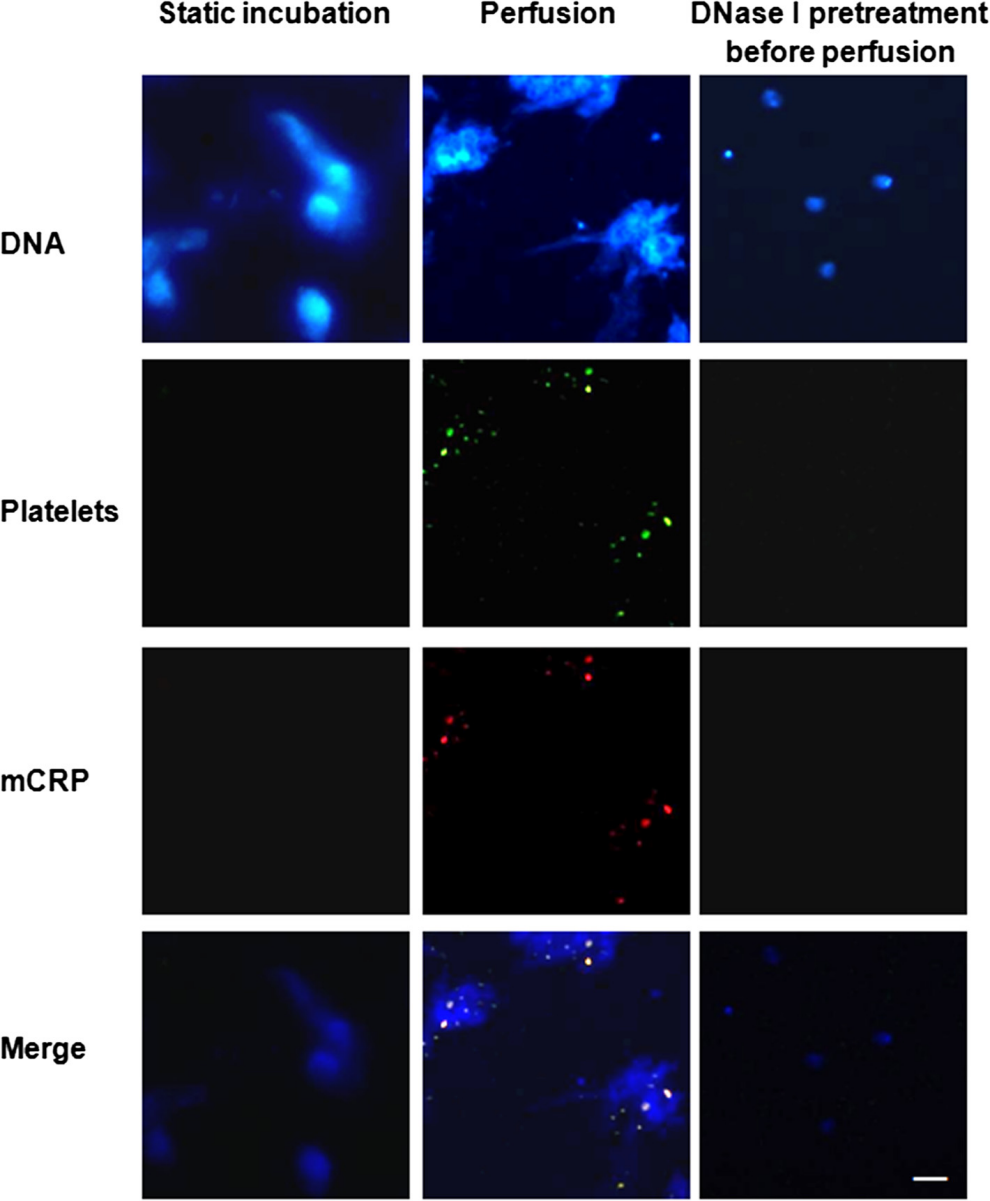
**Representative images of the depositions of platelets and mCRP on the immobilized ANCA-induced netting neutrophils (×100).** pCRP (60 μg/mL)-containing PRP was used in all groups. DNA was stained as a marker of netting neutrophils using DAPI (blue). Bright spots were neutrophils without NETs formation while cloud-like structures were NETs. Activated platelets were stained using FITC conjugated anti-CD62P mAb (green). Deposited mCRP was stained using Clone CRP-8 and TRITC-goat anti-mouse antibodies (red). All experiments were performed in triplicate. Scale bar: 50 μm.

It has been reported that NETs can offer a scaffold for platelets binding under flow conditions [16]. In the current study, when ANCA-induced netting neutrophils were coated on slides in flow chamber and were perfused with pCRP-containing PRP at 1500 s^−1^ for 10 min, activated platelets (CD62p positive) and deposited mCRP could be observed on slides. Co-localization analysis showed that CD62p positive platelets were mainly deposited on the DNA of NETs and mCRP was deposited on activated platelets. Static incubation of ANCA-induced netting neutrophils and pCRP-containing PRP for 10 min induced no deposition of activated platelets or mCRP, suggesting the activation of platelets relayed on the flow conditions, and the deposition of mCRP relayed on the activation of platelets. Pretreating the slides with DNase I digested the DNA and diminished the deposition of activated platelets and mCRP (Figure 2).

**Figure 3:**
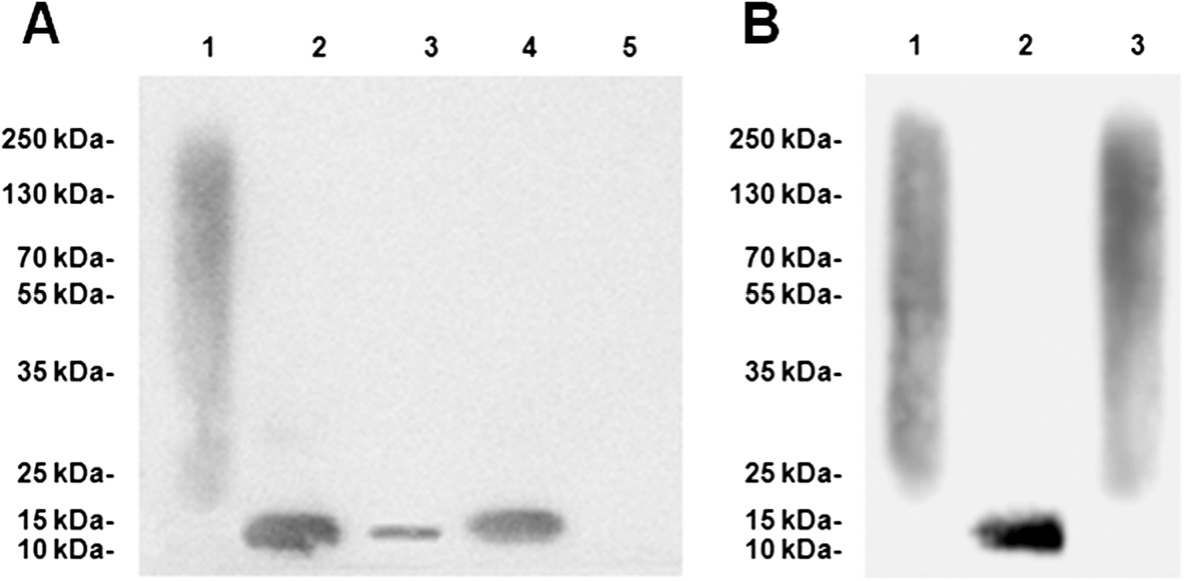
**Western-bolt with 1/20 of standard SDS. A**: Western-Blot assay of the CRP deposited on platelets. A typical result from 3 experiments was shown. pCRP control ran as a smear without a single band (lane 1), while mCRP control ran as a single band with a molecular weight of 15 kDa (lane 2). The substances deposited on slides ran as a 15 kDa single band without a smear after perfusion experiment with pCRP (60 μg/mL)-containing PRP (lane 3). A stronger band was detected after perfusion experiment with mCRP (60 μg/mL)-containing PRP (lane 4). The relative intensity of lane 4/lane3 was 2.45 ± 0.63. No smear or single band was detected after perfusion experiment with PRP which did not contain pCRP or mCRP (lane 5). **B**: Western-Blot assay of the co-incubation of isolated NETs and pCRP. A typical result from 3 experiments was shown. Lane 1: pCRP control. Lane 2: mCRP control. Lane 3: a smear without a single band was detected after pCRP was statically incubated with isolated NETs for 12 hr.

According to the study of Eisenhardt et al. [11], Clone CRP-8 recognizes both mCRP and pCRP, so Western-Blot with 1/20 of standard SDS was used to confirm it was mCRP not pCRP that was deposited on the CD62p positive platelets. After perfusion experiments, slides were rinsed gently with Hanks solution (containing Ca^2+^ and Mg^2+^) then the deposited substances were washed off vigorously with Hanks solution (containing Ca^2+^ and Mg^2+^) and were subjected to Western-Bolt with 1/20 of standard SDS. mCRP control (urea/EDTA denatured pCRP) ran as a single band with a molecular weight of 15 kDa which was close to its expected molecular weight, while pCRP control ran as a smear without a single band, suggesting partial dissociation occurred in Western-Bolt with 1/20 of standard SDS. If there was a mixture of mCRP and pCRP deposited on activated pletelets, both a smear and a single band should be detected in the Western-Bolt with 1/20 of standard SDS. As shown in Figure 3A, the substances deposited on slides migrated to a position similar to the mCRP control, suggesting it was mCRP not pCRP that was deposited on activated platelets after perfusion experiments.

In order to investigate whether NETs could directly dissociate pCRP into mCRP, pCRP was incubated statically with isolated NETs (without platelets) at 37°C for 12 hr. As shown in Figure 3B, static co-incubation with NETs did not dissociate pCRP into mCRP.

### mCRP does not bind directly on undigested NETs

**Figure 4:**
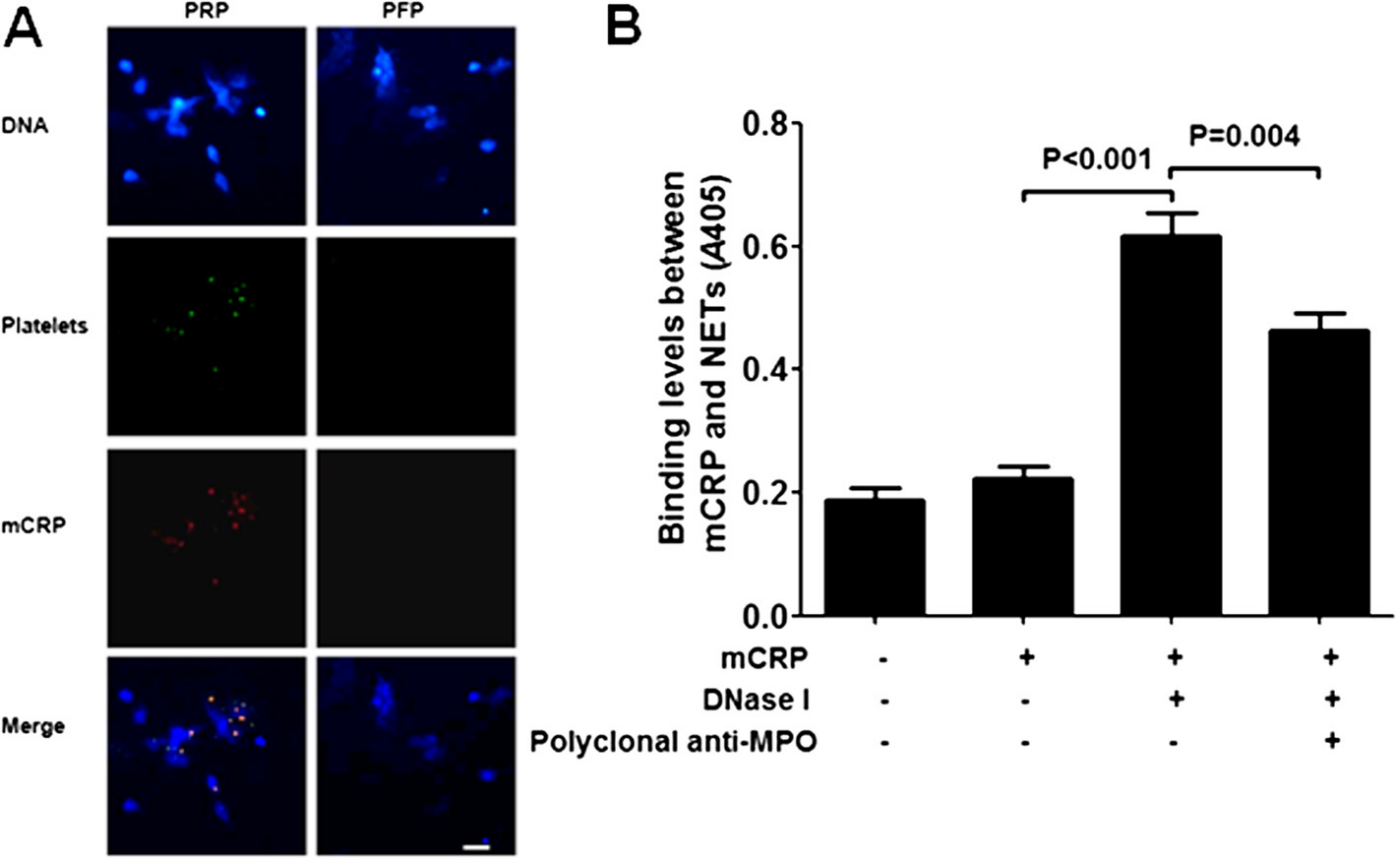
**mCRP does not deposit directly on NETs. A**: Representative images of the immunostaining of activated platelets and mCRP after ANCA-induced netting neutrophils were perfused with PRP or PFP (×100). Both PRP and PFP contained 60 μg/mL pCRP. All experiments were performed in triplicate. Scale bar: 50 μm. **B**: Results of ELISA of the binding between mCRP (60 μg/mL) and immobilized isolated NETs. Four wells of each group were analyzed and averaged.

MPO is an important component of NETs. We have reported that mCRP can bind MPO directly [24]. In order to further confirm the deposited mCRP in perfusion experiments did not bind NETs directly, immobilized ANCA-induced netting neutrophils were perfused with mCRP-containing PFP. As shown in Figure 4A, no deposition of platelets and mCRP on NETs could be detected after ANCA-induced netting neutrophils were perfused with mCRP-containing PFP. As shown in Figure 4B, mCRP did not deposit on the isolated NETs which were immobilized on plates (0.223 ± 0.035, expressed as *A* values at 405 nm) in ELISA analysis. However, when the immobilized NETs were pretreated with DNase I, increased binding of mCRP on the digested NETs was detected (0.617 ± 0.065, expressed as *A* values at 405 nm), suggesting some protein(s) contained in NETs should be the target of the mCRP binding. Polyclonal rabbit anti-MPO antibodies inhibited the binding between mCRP and the digested NETs (0.463 ± 0.049, expressed as *A* values at 405 nm). These results further demonstrated that although NETs contained MPO which could be bound by mCRP, undigested NETs could not be bound by mCRP directly. mCRP was only deposited on the activated platelets in perfusion experiments.

### mCRP deposited on the activated platelets during perfusion experiments enhances platelets activation

**Figure 5:**
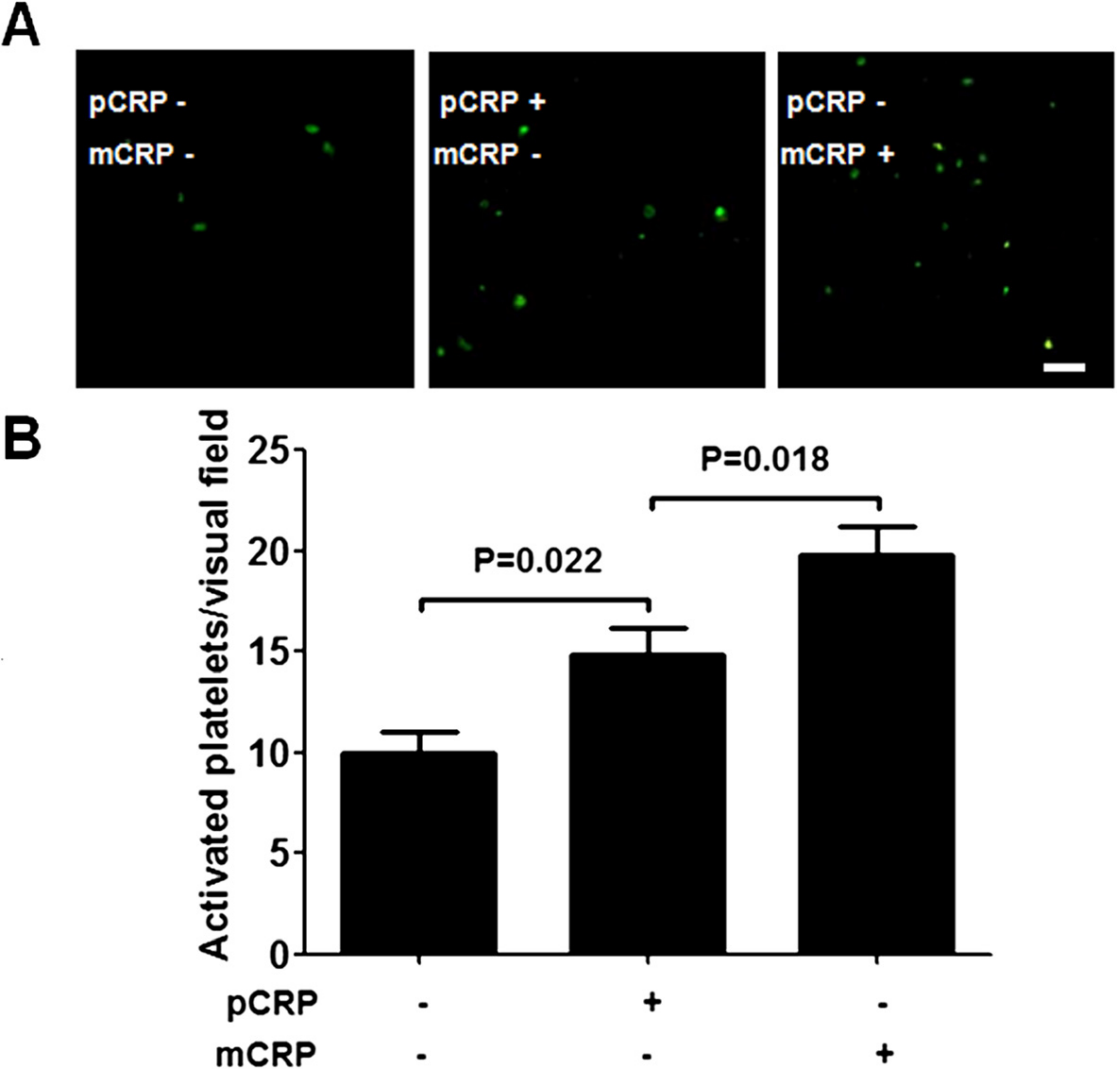
**Influence of the mCRP deposited during perfusion experiments on platelets activation. A**: Representative images of activated platelets (CD62p positive) after immobilized ANCA-induced netting neutrophils were perfused with pCRP-containing PRP or mCRP-containing PRP. The concentration of pCRP or mCRP was 60 μg/mL. Activated platelets were visualized using FITC conjugated anti-CD62P mAb (green). Scale bar: 50 μm. **B**: Calculated levels of the deposited CD62p positive platelets after immobilized ANCA-induced netting neutrophils were perfused with PRP which did not contain pCRP or mCRP, with pCRP-containing PRP or with mCRP-containing PRP. Results were expressed as activated platelets/visual field. Five visual fields of each group were analyzed.

As mentioned above, although static incubation with pCRP could not activate platelets, mCRP could generate from pCRP after perfusion experiments. Thus it was reasonable to speculate that under flow conditions, pCRP could enhance platelets activation through dissociating into mCRP. As shown in Figure 5, the number of activated platelets per visual field was 10.00 ± 2.23 after perfusing netting neutrophils with PRP (containing neither pCRP nor mCRP), while more activated platelets were detected after perfusing netting neutrophils with pCRP (60 μg/mL)-containing PRP (14.80 ± 3.11 activated platelets/visual field). PRP containing 60 μg/mL mCRP was used as the positive control (19.80 ± 3.19 activated platelets/visual field). These findings were confirmed by previous studies which reported that mCRP deposited on the surface of platelets significantly induced platelet adhesion further [25].

### mCRP enhances D-dimer formation in perfusion experiments

**Figure 6:**
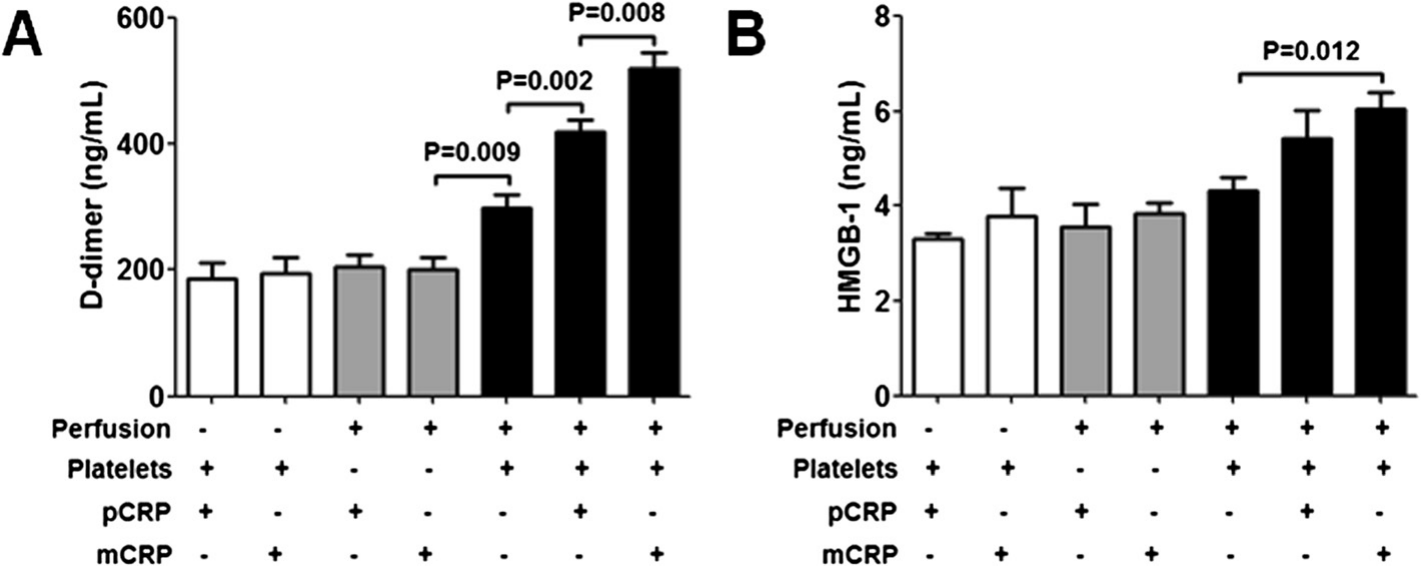
**Effects of mCRP on D-dimer formation and HMGB-1 secretion. A**: Effects of mCRP on the D-dimer formation. White columns: D-dimer levels of plasma after ANCA-induced netting neutrophils were statically incubated with pCRP-containing PRP or mCRP-containing PRP. Gray columns: D-dimer levels of plasma after ANCA-induced netting neutrophils were perfused with pCRP-containing PFP or mCRP-containing PFP. Black columns: D-dimer levels of plasma after ANCA-induced netting neutrophils were perfused with pCRP-containing PRP or mCRP-containing PRP. All experiments were performed in triplicate. **B**: Effects of mCRP on the HMGB-1 secretion of platelets. White columns: HMGB-1 levels of plasma after isolated NETs were statically incubated with pCRP-containing PRP or mCRP-containing PRP. Gray columns: HMGB-1 levels of plasma after isolated NETs were perfused with pCRP-containing PFP or mCRP-containing PFP. Black columns: HMGB-1 levels of plasma after isolated NETs were perfused with pCRP-containing PRP or mCRP-containing PRP. All experiments were performed in triplicate.

Previous studies have reported that immobilized NETs could provide a scaffold for *in vitro* thrombogenesis after perfusion with blood [16]. Since mCRP can enhance the activation of platelets, it should also be able to enhance the activation of coagulation and fibrinolysis system which is initiated by ANCA-induced netting neutrophils in perfusion experiments. To investigate whether the existence of mCRP showed a prothrombotic effect in perfusion experiments, the levels of D-dimer of plasma were measured after perfusion. As shown in Figure 6A, No increased levels of D-dimer could be detected after the immobilized ANCA-induced netting neutrophils were perfused with PFP, regardless of whether pCRP or mCRP was added to the system before perfusions. There was no increased D-dimer formation after the immobilized ANCA-induced netting neutrophils were incubated statically with PRP or PFP, regardless of whether pCRP or mCRP was added to the system. After the immobilized ANCA-induced netting neutrophils were perfused with PRP, increased levels of plasma D-dimer could be detected regardless of whether pCRP or mCRP was added to the system before perfusions, suggesting both coagulant and fibrinolytic systems were activated after perfusions. The existences of pCRP (60 μg/mL) or mCRP (60 μg/mL) in PRP further increased the levels of D-dimer in perfusion experiments. The levels of D-dimer after the netting neutrophils were perfused with mCRP-containing PRP was higher than that after the netting neutrophils were perfused with pCRP-containing PRP (520.0 ± 43.2 ng/mL *vs.* 420.3 ± 30.0 ng/mL, P = 0.008).

### mCRP enhances the HMGB-1 secretion of platelets in perfusion experiments

HMGB-1 can be released by activated platelets. Since HMGB-1 is able to activate neutrophils *in vitro* [20], it has been considered as a potential pathogenic factor which can accelerate the inflammation of AAV. To investigate whether mCRP could enhance the HMGB-1 secretion of activated platelets, the levels of HMGB-1 of plasma were measured after perfusion experiments. In order to exclude the interference of the HMGB-1 released by neutrophils, isolated NETs with the adjusted dsDNA concentration of 5 μg/mL were used to replace ANCA-induced netting neutrophils at the coating step. As shown in Figure 6B, No increased levels of HMGB-1 could be detected after isolated NETs were perfused with PFP, regardless of whether pCRP or mCRP was added to the system before perfusions. There was no increased HMGB-1 after the immobilized NETs were incubated statically with PRP or PFP, regardless of whether pCRP or mCRP was added to the system.

After the immobilized NETs were perfused with PRP (containing neither pCRP nor mCRP), the HMGB-1 level of plasma was 4.31 ± 0.49 ng/mL. After the immobilized NETs were perfused with pCRP (60 μg/mL)-containing PRP, the HMGB-1 level of plasma was 5.42 ± 1.05 ng/mL (P = 0.172, compared with the HMGB-1 level of plasma after the immobilized NETs were perfused with PRP which contained neither pCRP nor mCRP). After the immobilized NETs were perfused with mCRP (60 μg/mL)-containing PRP, the HMGB-1 level of plasma was 6.05 ± 0.61 ng/mL (P = 0.012, compared with the HMGB-1 level of plasma after the immobilized NETs were perfused with PRP which contained neither pCRP nor mCRP).

## Discussion

ANCA can induce the NETs formation of neutrophils and ANCA-induced netting neutrophils are present in patients with AAV [14]. As an important component of NETs, histones have been reported to be able to activate platelets directly and promote thrombin generation [15-17]. In the current study, undigested netting neutrophils could not activate platelets, while DNase I-pretreated netting neutrophils increased the expression of CD62p of platelets significantly in static incubation system. This phenomenon suggests that histones which are masked by the DNA of intact NETs are necessary for the activation of platelets in static incubation system. Contrary to the results obtained in static incubation system, the integrity of DNA is the prerequisite of the platelets activation in perfusion experiments, because shearing force is one of the key factors for platelets activation and only the intact DNA can offer a barrier for retaining platelets from flowing blood [25-27].

Although we could not exclude the possibility that pCRP also bound activated pletelets with immunofluorescence assay, Western-Blot with 1/20 of standard SDS helped us to distinguish whether the CRP deposited on activated platelets was mCRP, pCRP or the mixture of both mCRP and pCRP. Through this method, we demonstrated that the deposited CRP was mCRP not pCRP because it ran as a single band without a smear. Although after perfusion experiments, there was still some pCRP which did not dissociate into mCRP, no smear at higher molecular weights was detected in Western-Bolt. The reason should be that slides had been rinsed with Hanks solution (containing Ca^2+^) and the pCRP which did not bind platelets was removed before Western-Bolt.

It was noteworthy that static co-incubation of isolated NETs and pCRP without platelets did not generate mCRP. So it was not the components of NETs that dissociate pCRP in perfusion experiments. The existence of activated platelets should be the prerequisite of the formation of mCRP. According to previous studies, lysophosphatidylcholine exposed on the cell membrane of activated platelets can offer a surface for the dissociation of pCRP [9]. On activation and adhesion, platelets undergo a rapid change in morphology and composition of membrane lipids, leading to the exposure of bioactive lipid and the dissociation of adhered pCRP to mCRP [9].

pCRP and mCRP differ in their biological effects. mCRP can exert various effects that are distinct from those of pCRP. Intravenous pCRP administration significantly enhanced leukocyte rolling, adhesion, and transmigration via localized dissociation to mCRP, while inhibiting the dissociation of pCRP diminished these effects [27]. Several studies have demonstrated the important effect of mCRP on the activation of coagulation system [10,12]. Similar to the results of previous studies, we found that the platelets could be activated by mCRP in static incubation system. mCRP could even enhance the activation of platelets induced by DNase I-pretreated netting neutrophils in static incubation system. We also found mCRP deposited on the activated platelets could enhance the activation of platelets and the formation of D-dimer in perfusion experiments. So the activation of platelets in perfusion experiments with pCRP-containing PRP should be induced by two factors: shearing force and the deposited mCRP. To the best of our knowledge, our study is the first to show a causal role of mCRP on the thrombosis in AAV.

Although the expression of tissue factor in NETs has been reported by previous studies [28], we did not find the formation of D-dimer after the static co-incubation of netting neutrophils and PRP or after the perfusion of netting neutrophils with PFP. On the other hand, although the static co-incubation of mCRP and PRP could activate platelets, it did not induce the formation of D-dimer. Interestingly, the existence of mCRP could enhance the formation of D-dimer when the immobilized ANCA-induced netting neutrophils were perfused with PRP. Therefore, we think both flow conditions and platelets are necessary for the activation of coagulation system induced by ANCA-induced netting neutrophils, and the existence of mCRP should accelerate this process.

More and more studies demonstrate that inflammation and thrombosis can influence each other [18,19]. On one hand, inflammation has been suggested as a risk factor for VTE, on the other hand, apparent systemic inflammatory responses can be found in the acute phase of deep vein thrombosis [18,19]. HMGB-1 is an intracellular protein and function as a kind of proinflammatory mediator in AAV when released from cells. The levels of HMGB-1 in active AAV patients are significantly higher than those in remission [29-32]. Incubation of neutrophils with HMGB-1 significantly increases the NETs formation of neutrophils *in vitro* [20]. Interestingly, Wang et al. found that plasma levels of HMGB-1 correlated with circulating CRP level in AAV [32]. In the current study, we found that the existence of mCRP enhanced the HMGB-1 secretion of platelets under flow conditions. This indicates mCRP might be a potential link between thrombosis and inflammation in AAV.

We did not find direct binding of pCRP or mCRP to the activated neutrophils in perfusion experiments. This seems to be in contrast to the current literature [33]. We think there might be some reasonable explanations for this phenomenon. First, the duration of perfusion experiments (10 min) was not long enough. Second, before perfusion experiments, neutrophils had been incubated with ANCA and the Fc receptors on neutrophils, which were also the potential receptors for pCRP and mCRP, had been occupied by ANCA. Third, the NETs surrounded the activated neutrophils and prevented the binding of pCRP or mCRP on the cell surface.

In the current study, we also did not find the direct binding between mCRP and the undigested NETs, while NETs pretreated by DNase I could be bound by mCRP. Although this phenomenon could be explained by the binding between MPO and mCRP, it was noteworthy that polyclonal rabbit anti-MPO antibodies could not inhibit the binding totally (the *A* 405 nm was decreased by only 26%). This phenomenon means there might be some other components in NETs which can bind mCRP. Actually, previous studies indicate that mCRP has lectin-like properties and can bind galactose-containing residues [34-36]. So one possible explanation might be that mCRP also binds some substances in NETs which are full of galactose. This hypothesis needs further investigation.

The current study provides a potential link between mCRP and VTEs in AAV. Therefore, elevated circulating pCRP not only is a biomarker of AAV, but also plays an important role in the pathogenesis of AAV. Although our experiments are done *in vitro*, it is reasonable to speculate that all these processes might also occur *in vivo*. First, ANCA has been proved to be an important NETs inducer and enhanced formation NETs has been reported in patients with AAV [13]. Immunostaining in kidney tissue of humans with AAV demonstrated the existence of NETs [14]. The direct proof of the participation of NETs in the thrombogenesis in AAV has been reported. In the study of Nakazawa et al., NETs were identified in the thrombus in an AAV patient with deep vein thrombosis. The authors also found that the amount of NETs in the thrombus was significantly greater in that patient when compared with other thrombi unrelated to AAV [37]. Second, NETs have been proved to be able to induce apoptosis of endothelial cells directly *in vitro*, so the NETs induced by ANCA might injure the endothelial cells and offer a surface for the activation of coagulation system in AAV [38]. Third, the existence of mCRP in AAV has been proved by the previous study, which found that mCRP was observed in the cytoplasm of tubules and interstitium of renal biopsies from nine out of the ten patients with AAV [39].

There are some limitations of this study. First, no immunostaining of mCRP in the thrombus of patients with AAV was done and our understanding of the pathophysiology of pCRP dissociation was based on *in vitro* findings. Second, although hypercoagulable state is associated with the disease activity of AAV, the significance of the VTEs in AAV still needs further elucidation. Previous studies indicated the patients who had a VTE event seemed to be older than patients without VTE, but no differences about the indexes of disease severity were found between two groups [5]. It is noteworthy that all previous studies do not include a prospective screening protocol for VTEs and investigators count only clinically apparent VTEs for incidence estimates [1-5]. So the real incidence of VTEs in AAV and the influences of VTEs on the disease severity of AAV need to be investigated in future.

## Conclusions

In conclusion, ANCA-induced NETs can activate platelets and then prompt the formation of mCRP on activated platelets. Then the newly generated mCRP can further enhance the activation of platelets, the process of thrombogenesis, and the inflammatory response. So we think the increased circulating pCRP might be a potential therapy target in AAV and should be paid more attention.

## Abbreviations

AAV: Anti-neutrophil cytoplasmic antibody (ANCA)-associated vasculitis
ADP: Adenosine diphosphate
BSA: Bovine serum albumin
CRP: C-reactive protein
DAPI: 4’,6-diamidino-2-phenylindole
EDTA: Ethylene diamine tetraacetic acid
ELISA: Enzyme-linked immunosorbent assay
FITC: Fluorescein isothiocyanate
HMGB-1: High mobility group box 1
mCRP: Monomeric CRP
MPO: Myeloperoxidase
NETs: Neutrophil extracellular traps
PAGE: Polyacrylamide gel electrophoresis
PBS: Phosphate-buffered saline
pCRP: Pentameric CRP
VTE: Venous thromboembolism
PFP: Platelet-free plasma
PRP: Platelet-rich plasma
SDS: Sodium dodecyl sulfate
TBST: Tris buffered saline with Tween-20
TRITC: Tetraethyl rhodamine isothiocyanate
